# How Much Hypertension is Attributed to Overweight, Obesity, and Hyperglycemia Using Adjusted Population Attributable Risk in Adults?

**DOI:** 10.1155/2020/4273456

**Published:** 2020-08-14

**Authors:** Ebrahim Babaee, Arash Tehrani‐Banihashem, Babak Eshrati, Majid Purabdollah, Marzieh Nojomi

**Affiliations:** ^1^Student Research Committee, Preventive Medicine and Public Health Research Center, Iran University of Medical Sciences, Tehran, Iran; ^2^Preventive Medicine and Public Health Research Center, Department of Community Medicine, Iran University of Medical Sciences, Tehran, Iran; ^3^Department of Nursing, Faculty of Nursing, Tabriz University of Medical Sciences, Tabriz, Iran; ^4^Preventive Medicine and Public Health Research Center, Psychosocial Health Research Institute, Community and Family Medicine Department, School of Medicine, Iran University of Medical Sciences, Tehran, Iran

## Abstract

**Background:**

High blood pressure (HBP) is a proven risk factor for cardiovascular diseases. So, determining the extent of the contribution of the factors associated with HBP seems to be necessary. Accordingly, this study aimed to investigate how much the prevalence of HBP attributed to obesity and high blood glucose (HBG).

**Methods:**

Data were collected from 7612 participants extracted from a screening program in 2018, in Iran, which was conducted on the subjects with the age of 30 years old and older to investigate the prevalence of HBP and their associated risk factors. To collect data, we used a standard checklist in terms of the WHO STEPS manual, and a stratified multistage sampling method was also applied. The adjusted population attributable risk of overweight, obesity, and HBG for HBP was calculated by the logistic regression model using the aflogit module.

**Results:**

Among the studied people, 7.4% of male and 10.8% of female subjects were hypertensive. The adjusted analysis showed that, in men, 27% and 41% and, among women, 19% and 37% of HBP prevalence rates were attributable to obesity (BMI ≥ 30) and fast blood sugar (FBS) (≥126), respectively. In people with both obesity and HBG, 59% of the prevalence rate of HBP in men and 46% of the prevalence in women were due to the abovementioned risk factors altogether. The results show that, if obesity and HBG were eliminated, the prevalence of HBP could be theoretically decreased from 7.4% to 5.4% and 4.3% in male subjects and from 10.8% to 8.7% and 6.8% in female subjects, respectively.

**Conclusions:**

Our findings indicate that how much the prevalence of HBP attributes to obesity and HBG in middle-age and older population. It seems that the prevention programs should be administered in the general population, and excess body weight prevention programs should also be implemented in childhood.

## 1. Introduction

Abnormal arterial blood pressure is considered as hypertension or high blood pressure (HBP) [1]. The most popular type of hypertension is primary hypertension with a prevalence rate of ninety-five percent (95%) [2]. Several studies show that hypertension affects millions of people worldwide in terms of the risk of cardiovascular disease (CVD), in which the situation is getting worse.

Because different causes can lead to blood pressure in different patients, HBP is considered as a heterogeneous disorder [3]. Studies indicate that HBP is the result of an interaction among some genetics, metabolic, and behavioral factors such as obesity [4], blood sugar [5], and many other factors. Overweight and obesity, as anthropometric risk factors, have higher odds of hypertension [1].

Based on this evidence, diminishing excess weight is considered as a critical element in controlling HBP in both children and adults [6]. In adults, 75% of the incidence of HBP is directly due to obesity [7]. Moreover, determining that anthropometric and metabolic factors can affect blood pressure was examined in some previous studies; however, developing the preventive programs and also providing the related guidelines to control and decrease the harmful consequences of hypertension regarding the causing well-known risk factors are essential.

From the confirmed risk factors such as abnormal body mass index (BMI) [2] and high blood glucose (HBG) [5], which can raise the risk of blood pressure, few studies were performed with a preventive approach to determine that controlling the considered risk factors can how much reduce the risk of occurrence of hypertension. Moreover, previous studies have demonstrated that the effect of these two risk factors on blood pressure can be more prominent compared to the other known risk factors [8–10]. By considering all these, we conducted this cross-sectional study with a relatively large sample size to investigate how much the prevalence of hypertension is attributable to overweight, obesity, and HBG. Also, we investigated the theoretical prevalence of HBP in the sex-age subgroups (males +30 and females +30) if overweight, obesity, and HBG were eliminated.

## 2. Materials and Methods

This study was implemented in Tehran (Capital of Iran) in 2019. This study was performed in terms of the ethical guidelines and was then approved by the Research Council of Iran University of Medical Sciences.

The data were obtained from a screening program, and the aim was to estimate the prevalence of hypertension and their associated risk factors. The subjects of this study were the individuals with the age of 30 years old and older. A stratified multistage sampling method was applied to collect the study subjects. The participants were invited for screening in 16 health facility centers with the trained personnel, in Tehran and its suburbs. Finally, 7612 participants were included in this study. To collect the required information on HBP, BMI, blood sugar, and demographic characteristics such as age and sex, we used a standard checklist. Accordingly, this checklist was a measurement instrument according to the WHO STEPS manual.

### 2.1. Measurements

Measurement of anthropometric variables was conducted in a recommended manner. All the instruments were calibrated prior to use. To measure height and weight, the participants had light clothing with no shoes. Afterward, we applied the BMI index to evaluate obesity. Based on this index, the subjects were classified into four groups in terms of the WHO guideline as follows: BMI ranges are underweight, under 18.5 kg/m^2^; normal weight, 18.5 to 25; overweight, 25 to 30; and obese, over 30 [11, 12].

After resting for 15 minutes, blood pressure was measured for each person twice using the standardized mercury sphygmomanometers with a 10 to 15 min interval. The mean of these two measurements was considered as blood pressure, which was then recorded. In adults, systolic blood pressure equal to or less than 120 mm Hg and diastolic blood pressure equal to or less than 80 mm Hg were defined as a normal blood pressures. The systolic blood pressure equal to or more than140 mm Hg and/or the diastolic blood pressure equal to or more than 90 mm Hg were considered as HBP [13]. If the estimated mean blood pressure was equal to or above 140/90 mmHg, the subject was defined as hypertensive [14].

The American Diabetes Association guideline was applied for the measurement of fast blood sugar (FBS). The trained personnel performed the blood sugar test. FBS was measured by taking a venous blood sample after 12-hour overnight fasting. Moreover, the subjects with an FBS between 110 and 125 mg/dl, and an FBS equal to or more than 126 mg/dl were considered as impaired fasting glucose and diabetes, respectively [15].

The cases with hypertension and HBG (≥126) history with a performed treatment were diagnosed with hypertension and diabetes, respectively, regardless of their blood pressure and FBS levels during the screening program.

### 2.2. Statistical Analysis

Mean (±SD) and percentage were used to interpret the results for continuous and categorical variables by age and sex groups. The prevalence of overweight, obesity, and HBG was estimated by the age (+30) and sex groups. In addition, the prevalence of HBP was estimated by age-sex, BMI, and FBS groups, which was then tested using the chi^2^ statistical method.

We applied a logistic regression model using the aflogit module to estimate the adjusted population attributable risk fraction (PAR%) for the expected risk factors [16]. All the assumptions that were required for this model were also tested. The analysis and results were stratified by age-sex groups and then adjusted for all the factors under study.

The casual relationship between a risk factor(s) and a specific outcome can be inferred by an estimated PAR that specifies the intervention paths for prevention [17]. We examined the potential interaction term between BMI and FBS, and accordingly, no significant interaction was detected (*P*=0.940). By multiplication of the estimated prevalence of HBP by (1 – estimated PAR %), the theoretical prevalence rates of HBP were calculated after the elimination of overweight, obesity, and HBG. To estimate the adjusted odds ratios, the logistic regression model was applied. All the statistical analyses were performed at a 95% significance level using the STATA software version 14.

## 3. Results

In this study, 7612 subjects (48% men and 52% women) were included. The mean age of the participants was 47 ± 0.15 years old (ranged from 30 to 93 years old), and the majority of subjects (35.1%) were in the 30–39 age group in both sex. The prevalence of HBP was calculated as 7.4% (95% CI : (6.6, 8.3)) and 10.8% (95% CI : (9.8, 11.8)) in male and female subjects, respectively. About 37.4% (95% CI : (36, 38)) of the subjects (both sex) were overweight, 25.1% (95% CI : (24, 26)) were obese, and 6.1% (95% CI : (5.3, 7)) of them were diabetic (≥126). Findings indicated that 40% (95% CI : (37, 44)) and 17% (95% CI : (14, 22)) of the hypertensive cases were obese and diabetic, respectively. The detailed results of the demographic and anthropometric characteristics are reported in Table 1.

Calculated adjusted PAR% of HBP due to different categories of BMI, HBG, and obesity combined with HBG in the age-sex groups is presented in Table 2. The estimated PAR% due to BMI ≥ 30, BMI : 25–29.9, FBS ≥ 126, and FBS : 110–125.9 was 27%, 17%, 41%, and 30% in men over 30 years old and were also 19%, 9%, 37%, and 24% in women over 30 years old, respectively. These estimated PAR%s were nonsignificant. According to Table 2, HBP was significantly associated with BMI ≥ 30 (OR = 2.6 (*P* : <0.001)), BMI : 25–29.9 (OR = 3 (*P* : <0.001)), FBS ≥ 126 (OR = 2.9 (*P* : <0.001)), and FBS : 110–125.9 (OR = 2.4 (*P* : <0.001)) in male subjects over 30 years old and was also associated with BMI ≥ 30 (OR = 2.1 (*P* : <0.001)), BMI : 25–29.9 (OR = 2.2 (*P* : <0.001)), FBS ≥ 126 (OR = 2.5 (*P* : <0.001)), and FBS : 110–125.9 (OR = 2.1 (*P* : <0.001)) in female subjects over 30 years old.

As shown in Table 3, the prevalence rate of obesity (BMI ≥ 30) and diabetes (FBS ≥ 126) was 17.7% (95% CI : (16, 18)) and 5.2% (95% CI : (4, 6)) in male subjects over 30 years old and was also 33.9% (95% CI : (31, 34)) and 7.4% (95% CI : (6, 8)) in female subjects over 30 years old, respectively. The prevalence rates of the other categories of BMI and FBS are shown in Table 3.

About 0.7% (95% CI : (0.5, 1)) of men and 1.6% (95% CI : (1, 2)) of women were both obese and diabetic. The actual prevalence of HBP in male and female subjects over 30 years old was 7.4% (95% CI : (6.6, 8.3)) and 10.8 (95% CI : (9.8, 11.8)), respectively. Based on our findings, in male subjects, the theoretical prevalence of HBP was estimated as 5.4% (95% CI : (4.7, 6.2)) and 4.3% (95% CI : (3.7, 5.1)) by eliminating obesity (BMI ≥ 30) and HBG (≥126), respectively. In the following, the theoretical prevalence of HBP was estimated as 8.7% (95% CI : (7.9, 9.6)) and 6.8% (95% CI : (6.1, 7.6)) by eliminating obesity (BMI ≥ 30) and HBG (≥126) in female subjects, respectively. Also, eliminating both obesity and HBG combination changed the prevalence of HBP from 7.4% to 3% (95% CI : (2.5, 3.6)) in male and from 10.8% to 6.1% (95% CI : (5.4, 6.8)) in female subjects. Further estimations of the theoretical prevalence of HBP by eliminating other categories of BMI and HBG are reported in Table 3.

## 4. Discussion

In this study, we indicated how much hypertension is attributed to overweight, obesity, and hyperglycemia. Moreover, we evaluated the theoretical impacts of the elimination of different categorizes of excess body weight and HBG on the prevalence of HBP.

Our findings revealed that the prevalence of HBP in the subjects under study was 7.4% for men and 10.8% for women over 30 years old. By approving the results of previous studies [11, 18], in our study, the prevalence of HBP increased with the increase of age as a well-known risk factor. In addition, our results indicate that, theoretically, 27% of the prevalence of HBP in men and 19% in women over 30 years old were attributable to obesity. However, 17% of the prevalence of HBP in men and 9% in women over 30 years old were due to overweight. However, these calculated PAR%s were nonsignificant may be due to the low sample size in the subgroups. By concurrently considering diabetes and obesity, the estimated PAR% reached 59% and 46% in men and women, respectively. Accordingly, this highlights the importance of simultaneous control of these two risk factors in reducing the prevalence of HBP. In following, the effect of simultaneous control of these risk factors on the HBP reduction is further explained. Our findings were also in line with the results of previous studies performed in this regard. According to the results of the Framingham study, every 10 percent increase in body weight can increase the systolic BP level by about 5.6 mm Hg [19].

Based on the findings of the global burden of obesity study, the number of overweight and obese people will tremendously increase in 2030 and reach 1.35 billion and 573 million, respectively [20]. The studies conducted in Iran show that the prevalence of excess body weight and also obesity is on the rise in all the age and sex groups and the growth of obesity was rapid in Iran during recent years [20, 21]. In our study, the prevalence of overweight, obesity, and HBG was higher among women compared to men. Based on a national study (SuRFNCD-2007) in Iran which was conducted to determine the risk factors for noncommunicable diseases in 15–64 aged people, the prevalence of diabetes, hypertension, and obesity was 8.7%, 26.6%, and 22.3%, respectively. The study found that prevalence rates of diabetes, hypertension, and obesity were higher among females [22]. Our findings are in the line with the results of a systematic review conducted in Iran in 2014 [23], which reported that this difference could be due to the low physical activity in women.

Although the prevalence of HBP, obesity, and HBG was investigated in some previous studies in Iran, in terms of the prevention approach, it was unclear that eliminating the overweight, obesity, and HBG can how much reduce the HBP burden efficiently. Based on our findings, by the eliminating obesity, the theoretical prevalence of HBP decreased to 5.4% in men and 8.7% in women.

As stated earlier, our results revealed that notifiable percentages of both genders were obese. Also, 40% and 38% of the hypertensive cases were obese and overweight, respectively. In support of the previous studies, we showed that HBP is significantly associated with obesity both in men (OR : 2.6) and women (OR : 2.1). In our study that was conducted on middle-age and old people, we illustrated the beneficial effect of the elimination of excess body weight on the reduction of the prevalence of HBP. Several studies claimed that conducting interventions such as dietary intake improving, increasing physical activity, and reducing sedentary life style have effective impacts on the prevention of HBP by controlling excess body weight [6, 24].

In addition, our study indicated that 41% of the prevalence of HBP in men and 37% in women over 30 years old were theoretically attributable to HBG. Based on our results, in comparison to the excess body weight, the impact of HBG on the risk of occurrence of HBP is more notifiable; therefore, by eliminating obesity, the theoretical HBP prevalence could be decreased to 5.4% in male subjects and 8.7% in female ones. However, the elimination of HBG can reduce the HBP prevalence to 4.3% and 6.8% in men and women, respectively. Our findings showed that the importance of the effect of prediabetic status on increasing the prevalence of hypertension should not be overlooked. In this regard, 30% of the prevalence of HBP in men and 24% in women over 30 years old were due to the impaired blood glucose. Notably, diabetes should be considered as an important risk factor for HBP. Based on a study performed in 2013 in Iran, more than four million Iranian adults had diabetes, which has increased by 35% in recent years [25].

In the following, we indicated the impact of the concurrent elimination of both obesity and HBG on the decrease of the percentage of HBP, so that, by deleting these two risk factors combined in the cases, the prevalence of HBP reduced to 3% and 6.1% in men and women, respectively. In consistent with our results, more studies claimed that hypertension also has a well-established relationship with diabetes [26]. The results of these studies showed that diabetes is associated with two-fold increase of odds of being hypertensive [27], and the chance of occurrence of hypertension duplicates if FBS raises around 85.8 mg/dl [11]. In this regard, it is completely clear that controlling blood glucose can effectively affect the prevention of blood pressure raising.

It should not be overlooked that, besides excess body weight and HBG, some factors such as sodium intake and family history play an influential role in the occurrence of hypertension [27]. In support of these findings, our results revealed that weight loss and blood glucose controlling is moderately effective on hypertension.

Several studies suggest that extensive prevention programs should be aimed at the modification of both HBP and the associated risk factors in the target population. Although the prevalence of diabetes, hypertension, and obesity in Iran is not higher than in developed countries, the results are remarkable [22]. Despite the improved management and control of blood pressure, as well as the increased awareness in recent years, there has been no significant reduction in blood pressure [28]. Moreover, we must note that obesity and overweight are modifiable risk factors. It seems that conducting the prevention programs for overweight and obesity should be done in an early life period. Accordingly, that is the reason that more studies should be conducted to estimate PAF of these risk factors for HBP in children and teenagers to obtain sufficient information for conducting the preventive programs. However, a few studies were previously conducted on this issue in some countries. Many studies declared that, in comparison with different methods, population-based intervention is less costly and also more effective on managing HBP compared to other strategies, such as treating HBP among the obese or diabetic population [29].

### 4.1. Strengths and Limitations

Some strengths of our study were as follows: we conducted this study with a relatively high sample size, information for analysis was extracted from a screening program that was conducted in terms of the recommended guidelines, and compared to previous studies, we evaluated some further factors in estimating PAR.

Moreover, we also declare the weaknesses and some possible biases of this study. We had no data on dietary factors, physical activity, alcohol, family history, and other some risk factors for more investigations. Besides, the cases with hypertension, HBG history, and treatment history were considered as hypertensive and diabetic regardless of their blood pressure and blood glucose levels during the screening program. For this reason, we may be underestimated the association between blood pressure and blood sugar levels. Ultimately, despite these potential weaknesses, we hope that the results of our study be useful in health policymaking and in designing the prevention programs.

## 5. Conclusion

In this research, we determined how much we can reduce the prevalence of hypertension, theoretically in the population by eliminating excess body weight and lowering blood sugar level. This study also suggests that the prevention programs should be administered in the general population, regardless of the evaluated risk factors in this study. Despite the priority of the obesity, as a known problem, no comprehensive program currently exists to prevent or control obesity in Iran. These considerations should be followed at the shortest possible time in public health setting [30–32]. The results of this study can be useful in health policymaking and in conducting the prevention programs.

## Figures and Tables

**Table 1:** Characteristics of the study sample (*n* = 7612).

Variables	*n* (%)	Normotensive *n* = 6915^a^	Hypertensive *n* = 696^a^	*P* value
Gender				
Male	3657 (48.1%)	3387	270 (39%)	
Female	3954 (51.9%)	3528	426 (61%)	<0.001

Age group (yr.)				
30–39	2658 (35.1%)	2608	50 (7%)	
40–49	2293 (30.3%)	2173	120 (17%)	
50–59	1366 (18%)	1190	176 (26%)	
60–69	675 (8.9%)	507	168 (24%)	
≥70	583 (7.7%)	405	178 (26%)	<0.001

Body mass index (kg/m^2^)				
Normal weight (18.5–24.9)	2829 (37.5%)	2638	149 (22%)	
Overweight (25.0–29.9)	2823 (37.4%)	2566	257 (38%)	
Obese 1 (≥30)	1899 (25.1%)	1623	275 (40%)	<0.001

Fasting blood sugar (mg/dl)				
Normal (<100)	2691 (83.3%)	2446	245 (65%)	
Impaired fasting glucose (100–125)	343 (10.6%)	276	67 (18%)	
Diabetes (≥126)	198 (6.1%)	132	66 (17%)	<0.001

^a^The sum of subgroups may be less than total because of missing data.

**Table 2:** Adjusted population attributable risk and 95% confidence interval of HBP due to overweight, obesity, impaired blood sugar, and HBG, and estimated odds ratios for HBP among over 30-year-old population by sex groups.

Risk factor	Male +30		Female +30	
	Odds ratio^a^ (*P* value)	PAR%^a^ (95%CI)^b^	Odds ratio^a^ (*P* value)	PAR%^a^ (95%CI)^b^
BMI ≥ 30	2.6 (<0.001)	27 (−92, 72)	2.1 (<0.001)	19 (−56, 58)
BMI (25–29.9)	3 (<0.001)	17 (−87, 63)	2.2 (<0.001)	9 (−43, 41)
FBS ≥ 126	2.9 (<0.001)	41 (−25, 90)	2.5 (<0.001)	37 (−12, 82)
FBS (110–125.9)	2.4 (<0.001)	30 (−17, 82)	2.1 (<0.001)	24 (−98, 71)
BMI ≥ 30 & FBS ≥ 126	5.5 (<0.001)	59 (−29, 95)	3.2 (<0.001)	46 (−20, 90)

^a^Effect sizes adjusted for age, weight groups, and blood sugar groups; ^b^CI calculated on the log (1-AF) scale.

**Table 3:** Prevalence of risk factors and theoretical high blood pressure (HBP%) and 95% confidence interval if the related risk factor is eliminated among over 30-year-old population by sex groups.

Groups	Prevalence of risk factors (95% CI)					Actual HBP% (CI)	Theoretical HBP% if risk factor is eliminated (95% CI)				
	BMI ≥ 30	BMI (25–29.9)	FBS ≥ 126	FBS (110–125.9)	BMI ≥ 30 & FBS ≥126		BMI ≥ 30	BMI (25–29.9)	FBS ≥ 126	FBS (110–125.9)	BMI ≥ 30 & FBS ≥ 126
Male +30 yr (*n* = 3657)	17.7 (16, 18)	54.1 (52, 55)	5.2 (4, 6)	15.7 (13, 17)	0.7 (0.5, 1)	**7.4** (6.6, 8.3)	5.4 (4.7, 6.2)	6.1 (5.3, 6.9)	4.3 (3.7, 5.1)	5.2 (4.5, 5.9)	3 (2.5, 3.6)
Female+30 yr (*n* = 3954)	33.9 (31, 34)	71.8 (69, 72)	7.4 (6, 8)	18.9 (16, 20)	1.6 (1, 2)	**10.8** (9.8,11.8)	8.7 (7.9, 9.6)	9.8 (8.9, 10.8)	6.8 (6.1, 7.6)	8.2 (7.4, 9.1)	6.1 (5.4, 6.8)

## Data Availability

The data used to support this study are available from the corresponding author upon request.
